# Chemical synthesis of grafted cyclotides using a “plug and play” approach†

**DOI:** 10.1039/d4cb00008k

**Published:** 2024-04-29

**Authors:** Johannes Koehbach, Edin Muratspahić, Zakaria M Ahmed, Andrew M White, Nataša Tomašević, Thomas Durek, Richard J Clark, Christian W Gruber, David J Craik

**Affiliations:** a Institute for Molecular Bioscience, Australian Research Council Centre of Excellence for Innovations in Peptide and Protein Science, The University of Queensland Brisbane Queensland Australia koehbachjohannes@gmail.com d.craik@imb.uq.edu.au; b School of Biomedical Sciences, The University of Queensland Brisbane Queensland Australia; c Center for Physiology and Pharmacology, Institute of Pharmacology, Medical University of Vienna Vienna Austria; d Research School of Chemistry, Australian Research Council Centre of Excellence for Innovations in Peptide and Protein Science, Australian National University Australia

## Abstract

Cyclotides are a diverse class of plant-derived cyclic, disulfide-rich peptides with a unique cyclic cystine knot topology. Their remarkable structural stability and resistance to proteolytic degradation can lead to improved pharmacokinetics and oral activity as well as selectivity and high enzymatic stability. Thus, cyclotides have emerged as powerful scaffold molecules for designing peptide-based therapeutics. The chemical engineering of cyclotides has generated novel peptide ligands of G protein-coupled receptors (GPCRs), today's most exploited drug targets. However key challenges potentially limit the widespread use of cyclotides in molecular grafting applications. Folding of cyclotides containing bioactive epitopes remains a major bottleneck in cyclotide synthesis. Here we present a modular ‘plug and play’ approach that effectively bypasses problems associated with the oxidative folding of cyclotides. By grafting onto a pre-formed acyclic cyclotide-like scaffold we show that difficult-to-graft sequences can be easily obtained and can target GPCRs with nanomolar affinities and potencies. We further show the suitability of this new method to graft other complex epitopes including structures with additional disulfide bonds that are not readily available *via* currently employed chemical methods, thus fully unlocking cyclotides to be used in drug design applications.

## Abstract 2

‘Plug and play’ grafting – a simple and convenient modular synthesis method to access complex cyclotide grafts and generate potent GPCR ligands is described.

## Graphical abstract

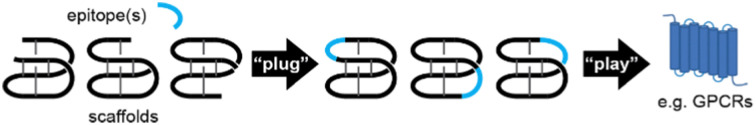

## Introduction

Over the last few decades, peptides have attracted increased interest for drug discovery and development approaches.^1–3^ Peptides, in principle, are able to fill the gap between larger protein-type therapeutics and traditional small molecules, but their use is still limited by challenges such as their typically low metabolic stability.^4^ Therefore, there is great interest in the stabilization of bioactive peptides *via* insertion (or grafting) into stable scaffolds, in particular into macrocyclic disulfide-rich peptides.^5^ Cyclotides, a versatile family of plant mini-proteins have emerged as such an attractive grafting scaffold. Cyclotides are characterized by a unique combination of a head-to-tail cyclic backbone and three intertwined disulfide-bridges that form a motif known as a cyclic cystine knot.^6^ Their tightly packed three-dimensional structures exhibit remarkable stability against chemical, thermal or enzymatical degradation.^7^ Importantly, kalata B1 (kB1), the prototypical cyclotide is amenable to a range of single amino acid mutations,^8,9^ but it can also accommodate insertions of so-called bioactive epitopes into one or more of its intercysteine loops.^10–16^ Epitope sequences up to 21 amino acids have been reported and resulting engineered cyclotides potently engage with a range of molecular targets.^10^ In particular, there are several examples of grafted cyclotides that target G protein-coupled receptors (GPCRs),^14,17–20^ one of the major classes of drug targets. However, cyclotide synthesis remains a major bottleneck and is limiting their widespread use.

**Fig. 1:**
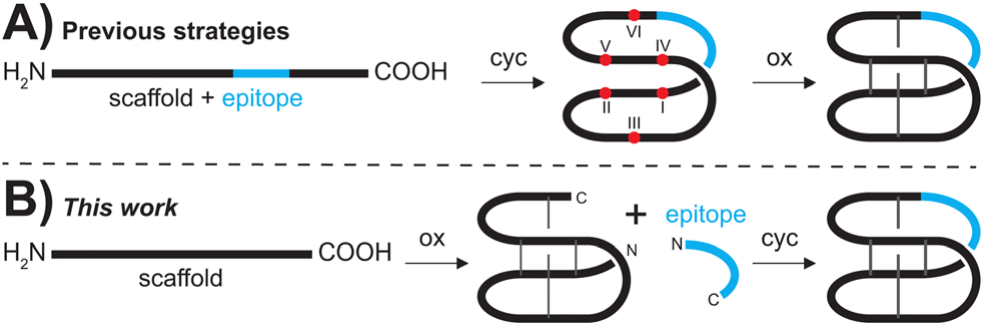
Comparison of current synthetic methodologies and the novel plug and play approach. (A) Various Fmoc-SPPS based methods start with the linear grafted cyclotide that is first backbone cyclized (cyc) and then oxidized (ox). (B) In the plug and play approach (this work), grafted cyclotides can be obtained by oxidizing the scaffold peptide prior to grafting of the epitope of interest to yield the backbone cyclized and oxidized product. Cysteine residues are numbered with Roman numerals, red dots represent free thiols and grey lines represent disulfide bonds.

*In planta*, cyclotides are ribosomally synthesized^21^ but they can be made *via* a variety of chemical strategies and are predominantly synthesized using Fmoc-based SPPS approaches.^22–27^ One of the most commonly applied protocols is the assembly of the peptide chain on a highly acid-labile resin to yield a fully side-chain protected peptide upon low-TFA cleavage of the peptide chain from the solid support. Backbone cyclization is achieved *via* in-solution ligation with standard coupling conditions using HATU/DIPEA.^28^ The crude cyclic peptide then undergoes side-chain deprotection prior to purification. Subsequent oxidative folding and another round of purification finally affords the desired product (Fig. 1A). Although recent improvements in automated SPPS have facilitated the assembly of so-called ‘problematic’ peptide sequences,^29^ access to grafted peptides by stepwise SPPS can be inefficient, resulting in low yields of crude peptides.

More importantly, oxidative folding of grafted peptides is often significantly impaired due to the inserted sequence causing structural perturbation that does not allow the formation of the native CCK fold,^8,9^ and affects yield and feasibility of grafting *via* traditional SPPS protocols (Fig. 1A).

To overcome the limitations of the chemical synthesis of cyclotide analogues we developed a modular ‘plug and play’ synthesis approach. We envisaged that bioactive epitopes could be added onto an already folded scaffold peptide (Fig. 1B), allowing reliable insertion of structurally diverse epitopes and thus rapid diversification of the scaffold. This approach circumvents two major bottlenecks of cyclotide synthesis, *i.e.*, (i) the stepwise assembly of longer and thus more difficult sequences and, more importantly, (ii) the often substantially impaired folding of grafted peptides.

## Results and discussion

**Fig. 2:**
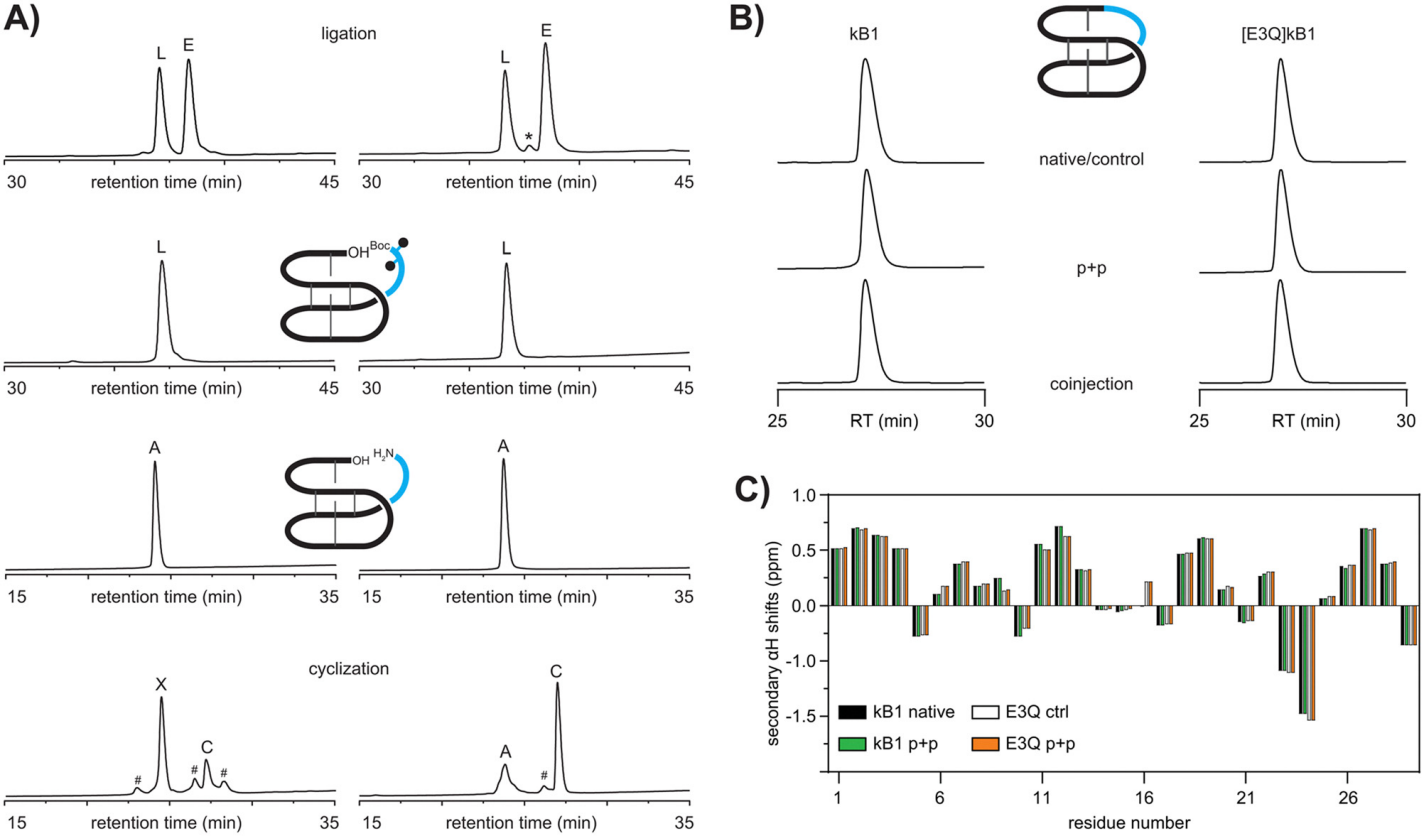
(A) Plug and play (p + p) synthesis of kalata B1 (left) and [E3Q]kB1 (right). Analytical comparison of cyclic kB1 and [E3Q]kB1 confirming identity of products as shown by retention time (RT) analysis (B) and αH chemical shift comparison (C). L ligated product, E excess epitope, A acyclic starting material, C cyclic product, X unwanted side-product, *peak showing *m/z* of epitope minus one Da, #no *m/z* observed, black dots indicate side-chain protecting groups.

To verify if a modular approach would work for the synthesis of cyclotides we attempted to synthesize the prototypic cyclotide kalata B1 (kB1) *via* a two-fragment approach. As proof-of-concept, we set out to synthesize the previously reported acyclic peptide, des(24–28)kB1,^30^ as a ‘receiver module’ and aimed to subsequently engraft the missing residues of the native loop 6, *i.e.* RNGLP into that sequence. The acyclic precursor des(24–28)kB1 (hereafter referred to as ΔL6kB1) was successfully assembled *via* automated Fmoc-SPPS, folded and purified as previously described^31^ (Fig. S1, S2 and S11, ESI†). We then envisaged a two-step ligation protocol would be required to avoid self-cyclization of two peptide fragments with free amino and carboxy termini resulting in self-cyclization of the individual reaction partners. Using Fmoc-SPPS on hyper acid-labile 2-chlorotrityl chloride resin, we generated a fully side-chain and N-terminally protected version of loop 6, *i.e.*, Boc-R(Pbf)N(Trt)GLP-OH (Boc-L6) to rule out self-cyclization and unwanted side reactions of the epitope. To further reduce the possible self-cyclization of the fully unprotected scaffold ΔL6kB1 and favor ligation of the two fragments, we used a four-fold molar excess of the epitope Boc-L6. The reaction between folded ΔL6kB1 and Boc-L6 (1 mM scaffold, 4 : 4 : 8 equivalents epitope : HATU : DIPEA) yielded the desired ligated peptide as a major product (67.8% isolated yield) (Fig. 2A) and without any evidence for self-cyclization of the scaffold ΔL6kB1. LCMS analysis did not indicate racemization of the C-terminal proline and showed only the expected ligated product and excess epitope. Both peptides were readily recovered during subsequent HPLC purification of the ligation reaction. Next, we deprotected the linear peptide applying standard side-chain deprotection conditions (TFA : TIPS : H_2_O, 90 : 5 : 5). The reaction proceeded efficiently and allowed recovery of fully deprotected linear kB1 (Fig. 2A). Finally, backbone cyclization was attempted by treating the linear peptide (1 mM) with HATU/DIPEA (1 : 2 eq.) in DMF for one hour followed by HPLC purification. Analysis of the crude reaction after one hour revealed two major products, both exhibiting the expected *m/z* of the desired product (Fig. 2A). Additional minor peaks present in the cyclization reaction could not be identified by LCMS analysis. Further characterization using carboxypeptidase Y digest revealed the early eluting major product (63%) to be an unwanted self-cyclization product between the free N-terminal amino group of arginine 24 and the side-chain carboxy group of the glutamic acid residue 3 (Fig. S6, ESI†). The minor product (27%) was subjected to analysis using analytical HPLC as well as NMR. Co-injection of authentic kB1 or synthetic samples resulted in a sharp single peak on HPLC (Fig. 2B). Similarly, secondary αH NMR chemical shifts of authentic kalata B1 and the late eluting minor product obtained from the cyclization reaction were identical, thus unambiguously confirming identity of the two samples (Fig. 2C).

Having established a successful plug and play methodology, we set out to optimize the scaffold for subsequent grafting applications. Since the presence of the glutamic acid residue seemed to favor formation of an unwanted side-product, and thus dramatically reduced yields of the desired cyclic product, the next logical step was to investigate possible substitutions of the glutamic acid residue. The full-length backbone cyclic version of [E3A]kB1 was previously shown to give reasonable folding yields, ∼50% compared to native kB1.^8^ However, concomitant removal of loop 6 appears to significantly perturb the folding of the peptide ΔL6[E3A]kB1, and only minor amounts (∼4%) of putatively correctly folded peptide were observed (Fig. S1, ESI†). We hypothesized that a more conservative replacement of the glutamic acid with glutamine might still be able to provide sufficient hydrogen bonding interactions to stabilize the native fold within the linear ΔL6kB1 sequence. The same folding conditions used for ΔL6kB1 also yielded correctly folded ΔL6[E3Q]kB1, albeit with the putatively correctly folded peptide being only ∼10% and ∼15% being a putative dimer. We further investigated whether different buffers and the repeated addition of fresh redox reagents could help to increase folding yields.^32^ Indeed, fresh addition of oxidized glutathione every 24 hours helped to increase folding yields up to 16.6% over 96 hours and final optimized conditions afforded the desired product in isolated yields of >15% with only traces of dimer present (<2%) as judged by RP-HPLC (Fig. S1, ESI†). Isolated oxidized ΔL6[E3Q]kB1 was then used to proceed with the plug and play methodology as described for kB1. Ligation of ΔL6[E3Q]kB1 (1 mM) with 4 equivalents of Boc-L6 and HATU : DIPEA (4 : 8 eq.) in DMF for one hour as well as subsequent deprotection of the purified product using TFA : TIPS : H_2_O 90 : 5 : 5 for one hour both proceeded smoothly (53% and 94% isolated yields), and the purified linear deprotected peptide was subjected to final backbone cyclization for one hour using HATU : DIPEA (1 : 2 eq.) in DMF. The major product (49% isolated yields) was a late eluting peak exhibiting the desired *m/z* corresponding to cyclic [E3Q]kB1. The peptide co-eluted with a sample that was obtained *via* a previously reported strategy^28^ confirming successful synthesis of [E3Q]kB1 *via* our plug and play method (Fig. 2B). Secondary chemical shift analysis confirmed that the mutation and chemical strategy does not disrupt the native structure of kB1 (Fig. 2C).

To verify the suitability of our methodology for cyclotide grafting, we synthesized G protein-coupled receptor ligands. Following on from our recent work of grafting the endogenous *κ*-opioid receptor ligand dynorphin A (1–13) (YGGLFRRIRPKLK, hereafter referred to as dynA) onto the cyclic scaffold SFTI^33^ we were interested to determine the effect of a different scaffold on biological activity of this epitope sequence. As our previous attempts to graft dynA onto kB1 using currently established protocols^26,28^ were unsuccessful we set out to synthesize the N-terminally protected version of dynA and apply our plug and play strategy. We envisaged that besides protection of the N-terminal amino group using Boc for the initial ligation reaction, a selective protection of any side chain amino group would be necessary for both ligation and cyclization reactions. Thus, lysine residues protected with 1-(4,4-dimethyl-2,6-dioxocyclohex-1-ylidene)-3-methylbutyl (ivDde) were used to prevent unwanted side product formation during these steps. Automated Fmoc-SPPS of linear dynA was successful and HPLC purification yielded the fully protected peptide Boc-Y(*t*Bu)GGLFR(Pbf)R(Pbf)IR(Pbf)PK(ivDde)LK(ivDde)-OH.

As for kB1 and [E3Q]kB1 all steps proceeded smoothly, and we were able to obtain the full-length cyclic dynA grafted into loop 6 of [E3Q]kB1 with an overall yield of 17.4% over three steps (Fig. S4, ESI†). We then tested the peptide using competitive radioligand binding assays as well as functional activation using cAMP inhibition assays. ΔL6[E3Q]kB1-dynA showed low nanomolar affinity and full agonist activity at the *κ*-opioid receptor (Table 1) that is comparable to the recently published peptide helianorphin-19.^33^

**Table 1:** Pharmacological data of [E3Q]kB1-dynA grafts at the *κ*-opioid receptor

Peptide	*K*_*i*_ (nM)	EC_50_ (nM)	*E*_max_ (%)
Dynorphin A 1–13	n.d.	3.4 ± 0.9	100
ΔL6[E3Q]kB1-dynA	6.2 ± 4.2	68.8 ± 23.1	139.4 ± 20.2
ΔL3[E3Q]kB1-dynA	1.4 ± 0.9	28.0 ± 9.8	109.0 ± 27.4
ΔL5[E3Q]kB1-dynA	3.2 ± 1.7	112.3 ± 68.2	128.0 ± 21.0

Encouraged by these data, we explored the impact of grafting dynA into various other loops of the cyclotide scaffold. Since acyclic versions of kB1 that have been linearized in loops 2, 3, 5 and 6 have been shown to fold into a native cyclotide-like structure^31^ we synthesized linear versions of [E3Q]B1 without loops 2, 3 and 5 respectively, leaving only a single flanking amino acid on either side of the cysteine (Table S1 and Fig. S2, S11, ESI†). All peptides were subjected to ‘standard’ as well as the ‘optimized’ folding conditions determined for ΔL6[E3Q]kB1. Optimized conditions allowed us to obtain sufficient material for all linear ΔL#[E3Q]kB1 variants (Table S1 and Fig. S1, S2, ESI†). While our plug and play methodology allowed us to successfully graft dynA in loop 3 (11.2% yield) and loop 5 (7.1% yield), we were unable to introduce dynA into loop 2. ΔL3[E3Q]kB1-dynA and ΔL5[E3Q]kB1-dynA were assessed in binding and functional activity assays and were found to exhibit nanomolar affinities at KOR with full agonist activity (Table 1).

To further explore the suitability of our newly established method for grafting of GPCR ligands and to determine the scope of epitopes that can be introduced, we then attempted to graft other structurally complex sequences. Notably, the grafting of sequences containing additional disulfide-bonds has not been previously reported for the kalata scaffold. Hence, we aimed to introduce octreotide into loop 6 of our receiver peptide ΔL6[E3Q]kB1. Octreotide, an eight amino acid analog of natural somatostatin is a high affinity ligand for the somatostatin receptor 2 (SST_2_R) and is clinically used to treat acromegaly.^34^ It contains one disulfide bond and two d-amino acid residues. Linear protected octreotide was synthesized as Boc-fC(Acm)Fw(Boc)K(ivDde)T(*t*Bu)C(Acm)T(*t*Bu)-OH. After formation of the intramolecular disulfide bond, oxidized octreotide was then grafted onto ΔL6[E3Q]kB1. All steps proceeded smoothly, and cyclic ΔL6[E3Q]kB1-octreotide (Table S3 and Fig. S5B, ESI†) was successfully obtained (16.1% yield). We then tested for activity on cells expressing SST_2_R using the PRESTO-Tango assay.^35^ ΔL6[E3Q]kB1-octreotide showed agonist activity with an EC_50_ of 1.5 ± 0.6 μM and an *E*_max_ of 82.1 ± 14.2% compared to octreotide (Fig. S7, ESI†). Next, we aimed to graft the endogenous oxytocin/vasopressin receptor ligands oxytocin and vasopressin as well as the synthetic complement receptor 5a ligands BM213 and BM221^36^ onto our scaffold ΔL6[E3Q]kB1. Synthesis of all peptides was successful (Table S3 and Fig. S5B, ESI†), but none of the compounds exhibited agonistic activity (Fig. S8 and S9, ESI†).

Having successfully applied our novel methodology to access various cyclotide grafts as GPCRs ligands, we then “played” and synthesized further grafted peptides to target other proteins. Of particular interest, was to further demonstrate the suitability of our method to generate grafts that may not be readily accessible by currently used synthetic strategies, in particular epitopes containing additional disulfide-bonds. Therefore, we synthesized a fully protected version of the trypsin inhibition loop (Boc-GC(Acm)T(*t*Bu)K(ivDde)S(*t*Bu)IPPIC(Acm)G-OH) of SFTI and successfully grafted the disulfide bonded epitope onto ΔL6[E3Q]kB1 to yield ΔL6[E3Q]kB1-SFTI (18.7% yield, Table S3 and Fig. S5A, ESI†). Glycine residues flanking cysteines were introduced as spacer residues. The peptide retained weak activity with ∼45% inhibition of trypsin at 16.6 μM (Fig. S10, ESI†). Finally, we attempted to graft the active turn region of the conotoxin MrIA (synthesized as Boc-GC(Acm)GY(*t*Bu)K(ivDde)LC(Acm)G-OH) onto our receiver peptide ΔL6[E3Q]kB1. MrIA is a *χ*-conopeptide with inhibitory activity at the neuronal norepinephrine transporter (NET).^37^ While synthesis of ΔL6[E3Q]kB1-MrIA proceeded without problems (18.2% yield, Table S3 and Fig. S5A, ESI†), the peptide did not inhibit NET at concentrations up to 100 μM. While these two examples only showed weak or no activity, their synthesis was readily accomplished.

## Conclusions

In summary, we have established a convenient and robust approach to rapidly access a structurally diverse range of grafted cyclotides that fully circumvents the problems associated with the oxidative folding of non-native CCK peptides. One risk of our novel strategy is the potential of epimerization during amide bond formation between unprotected amino acids, particularly during the final cyclization step. While no evidence for epimerization was observed for shown examples, this might be highly sequence and residue specific, and needs to be carefully analyzed. The use of different coupling reagents (*e.g.*, DIC/Oxyma) may reduce this risk and introduction of C-terminal glycine residues may fully circumvent epimerization problems. We show that this strategy allows access to peptides with nanomolar affinities and potencies at GPCRs, that have previously been unable to be obtained *via* currently used methods. We further show this method to be able to easily introduce complex epitopes, including sequences containing additional disulfide bonds or non-standard amino acids into different loops of the cyclotide scaffold. Thus, our approach further unlocks the potential of cyclotides in drug design applications for a variety of pharmaceutically relevant targets.

## Author contributions

D. J. C. and J. K. conceived the project. J. K. and Z. M. A. synthesized and characterized peptides. J. K., E. M., Z. M. A. and N. T. performed *in vitro* pharmacological assays. A. M. W. and T. D. analysed NMR data. R. J. C., C. W. G. and D. J. C. acquired funding. J. K. wrote original manuscript draft. T. D., C. W. G., A. M. W., R. J. C. and D. J. C. edited draft. All authors read and approved the final manuscript.

## Conflicts of interest

There are no conflicts to declare.

## Supplementary Material

CB-005-D4CB00008K-s001
