# Supplementary material for: Chemical synthesis of grafted cyclotides using a “plug and play” approach
Source: RSC Chem Biol. 2024 Apr 29;5(6):567–71. doi: 10.1039/d4cb00008k (PMC11151825; doi:10.1039/d4cb00008k)
Supplement: CB-005-D4CB00008K-s001 [file CB-005-D4CB00008K-s001.pdf]

Electronic Supplementary Material (ESI) for RSC Chemical Biology.
This journal is © The Royal Society of Chemistry 2024

Supplementary Information

Chemical synthesis of grafted cyclotides using a "plug and play" approach

Johannes Koehbach*, Edin Muratspahić, Zakaria M Ahmed, Andrew M White, Nataša Tomašević, Thomas Durek, Richard J Clark, Christian W Gruber and David J Craik*

*Correspondence to:

j.koehbach@uq.edu.au

d.craik@imb.uq.edu.au

Table of Contents

Section 1: Peptide synthesis, cleavage, and RP-HPLC purification 3

Section 2: Oxidative folding and characterization of scaffold peptides 4

Section 3: Oxidation and characterization of protected epitope peptides 7

Section 4: 'plug and play' grafting and characterization of cyclic peptides 10

Section 5: Carboxypeptidase Y digest and MALDI-TOF MS 14

Section 6: KOR pharmacology 15

Section 7: PRESTO-Tango assay 16

Section 8: pERK assay 18

Section 9: Trypsin inhibition assay 19

Section 10: NMR characterization 20

References 21

Section 1: Peptide synthesis, cleavage, and RP-HPLC purification

Peptides were synthesized by automated Fmoc SPPS on a Symphony peptide synthesizer (Protein Technologies) or a CS136X synthesizer (CS Bio). Following side-chain protecting groups were used: Cys(Trt), Cys(Acm), Glu(tBu), Lys(ivDde), Asn(Trt), Gln(Trt), Arg(Pbf), Ser(tBu), Thr(tBu), Trp(Boc) and Tyr(tBu). Peptides were assembled on 2-CTC resin (substitution value 0.45 or 1 mmol/g, 0.125 mmol synthesis scale). The first amino acid was coupled to the resin for 1 h in Dichloromethane (DCM), with a 2-fold excess of amino acid and 8-fold excess of N,N-Diisopropylethylamine (DIPEA). After coupling the resin was treated three times with a mixture of Methanol:DCM:DIPEA (17:2:1) for one minute each to cap any remaining reactive functionalities on the resin. Fmoc deprotection was carried out with piperidine in DMF (20% (v/v)) twice for two minutes. Subsequent amino acids were coupled twice for 10 minutes using an amino acid/2-(6-chloro-1-H-benzotriazol-1-yl)-1,1,3,3-tetramethylaminium hexafluorophosphate (HCTU)/DIPEA ratio of 4:4:8 relative to resin loading. DMF was used for resin washing between all steps. After chain assembly was completed, the resin was washed with DCM and dried under nitrogen.

For concomitant cleavage of the peptide chain and side-chain removal, the dry resin was then treated with TFA:TIPS:H2O (90:5:5, v/v/v) for two hours, excess TFA was removed, and the peptides precipitated in ice-cold diethyl ether. The precipitate was filtered and dissolved in a 50:50 mixture of solvent A (0.1% TFA in water) and solvent B (90% acetonitrile, 0.1% TFA in water). N-terminally Boc- and side-chain protected peptides were obtained by treating the resin ten times with 1% TFA in DCM for four minutes each. A 50:50 mixture of solvent A and B was added, and DCM removed on a rotary evaporator. Crude peptides were then lyophilized prior to purification by RP-HPLC.

Samples were purified using preparative and semipreparative RP-HPLC on a Shimdazu Prominence HPLC system on Phenomenex Gemini columns (5 µm C18 110 Å, 250 x 21.2 or 10 mm respectively). Correct products were identified using ESI-MS on a Shimadzu 2020 or a SCIEX API2000 mass spectrometer. Purity of all compounds was assessed either by UPLC analysis using a Luna Omega C18 column (Phenomenex 1.6 µm, 100 Å, 50 x 2.1 mm) with a linear gradient of 1 to 61% B over 15 minutes on a Shimadzu Nexera UPLC system or analytical HPLC using an Agilent Zorbax column (Agilent 150 x 2.1 mm) on a Shimadzu Prominence HPLC system. A linear gradient of 5 to 80% solvent B over 35 minutes was used for unprotected peptides, for protected peptides a linear gradient of 50 to 100% solvent B over 30 minutes was used.

Section 2: Oxidative folding and characterization of scaffold peptides

Oxidation of peptides (Table S1) was achieved by dissolving peptides at a concentration of 0.2-0.5 mg/mL in oxidation buffer (different ratios of 0.1 M NH_4HCO_3 and 2-propanol, pH 8.2-8.5, varying amounts of reduced and/or oxidized glutathione). Oxidized peptides were purified after overnight incubation (>18 hours at room temperature or 4 ^oC). Initial folding buffer conditions: 0.2-0.5 mg/mL peptide in 50% 0.1 M NH_4HCO_3, 50% 2-propanol, 1mM GSH, pH ~8.5, 20 ^oC for 24 hours. Final optimized conditions: 0.2-0.5 mg/mL peptide in 20% 0.1 M NH_4HCO_3, 80% 2-propanol, 1mM GSSG, pH ~8.5, 4 ^oC for 48-96 hours.

Table S1. Linear oxidized scaffold peptides

**Peptide**	**Sequence**[a]	**Mass (Da)**[b]		**yield (%)**[c]
		**calculated**	**observed**	
ΔL6kB1	VCGETCVGGTCNTPGCTCSWPVCT	1186.5 $[M+2H]^{+2}$	1186.8 $[M+2H]^{+2}$	15.4/23.4
ΔL6[E3A]kB1	VCGATCVGGTCNTPGCTCSWPVCT	1157.5 $[M+2H]^{+2}$	1157.5 $[M+2H]^{+2}$	3.8 (0.6)/4.6 (0.8)
ΔL6[E3Q]kB1	VCGQTCVGGTCNTPGCTCSWPVCT	1186.0 $[M+2H]^{+2}$	1186.2 $[M+2H]^{+2}$	9.4 (15.1)/16.1 (1.9)
ΔL2[E3Q]kB1	TCNTPGCTCSWPVCTRNGLPVCGQTCV	1397.6 $[M+2H]^{+2}$	1397.9 $[M+2H]^{+2}$	3.7/8.1
ΔL3[E3Q]kB1	GCTCSWPVCTRNGLPVCGQTCVGGTCN	1355.6 $[M+2H]^{+2}$	1355.9 $[M+2H]^{+2}$	2.6/9.5
ΔL5[E3Q]kB1	VCTRNGLPVCGQTCVGGTCNTPGCTCS	1313.0 $[M+2H]^{+2}$	1313.4 $[M+2H]^{+2}$	27.0/30.5

[a]All cysteines are oxidized, all peptides were synthesized as C-terminal acids

[b]Represents monoisotopic mass of fully oxidized peptides

[c]Yields from oxidation using initial and optimized conditions. Yields of putative dimer are given in brackets. Yields calculated by automated integration of the area under the curve from 5-15 minutes as shown in Fig. S1

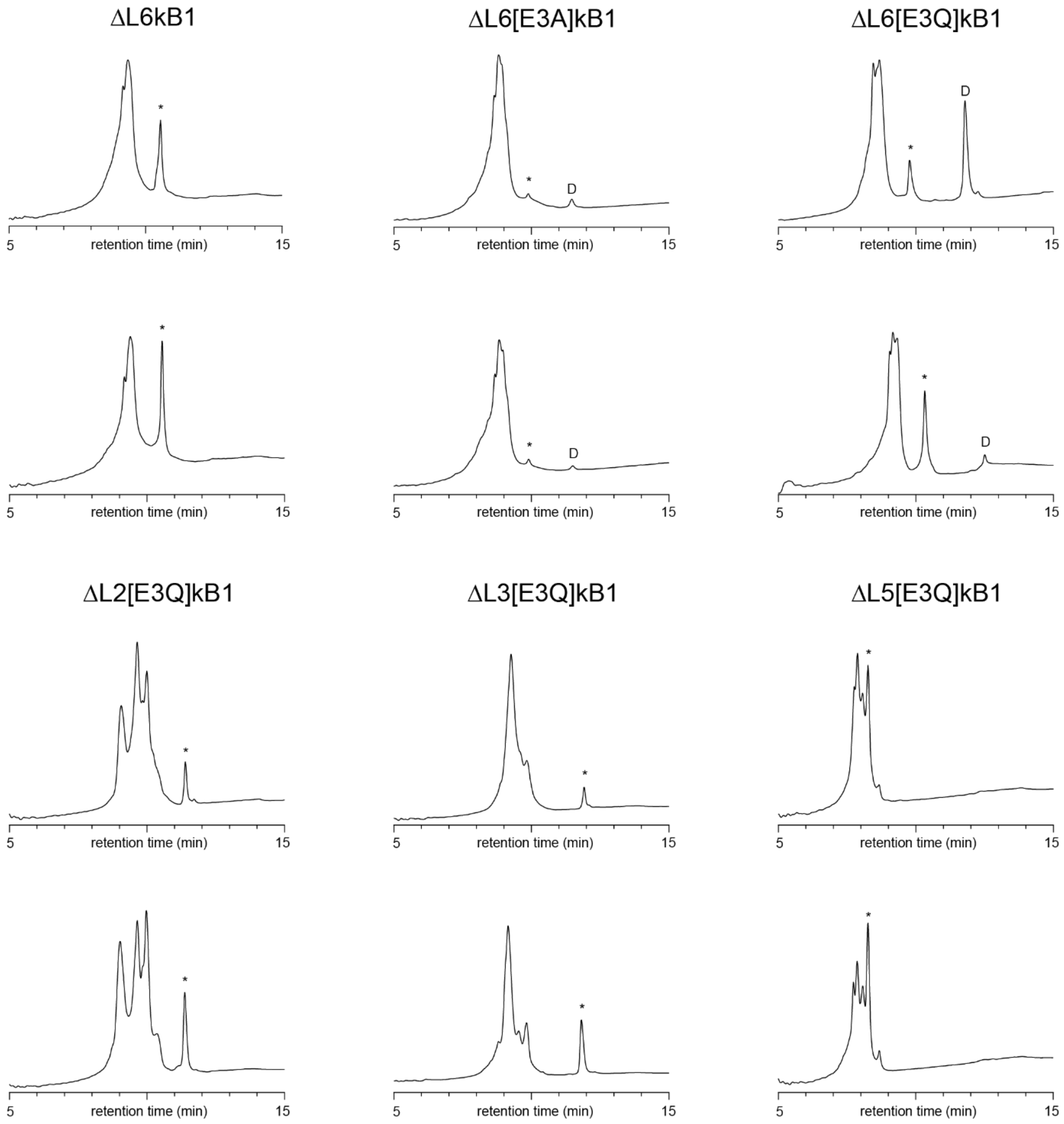

Fig. S1 Comparison of folding of linear kB1 scaffold peptides. 'Initial' conditions (top panels) versus 'optimized' condition (bottom panels) for each scaffold. * denotes native fold, D denotes putative dimer, see Table S1 for calculated yields

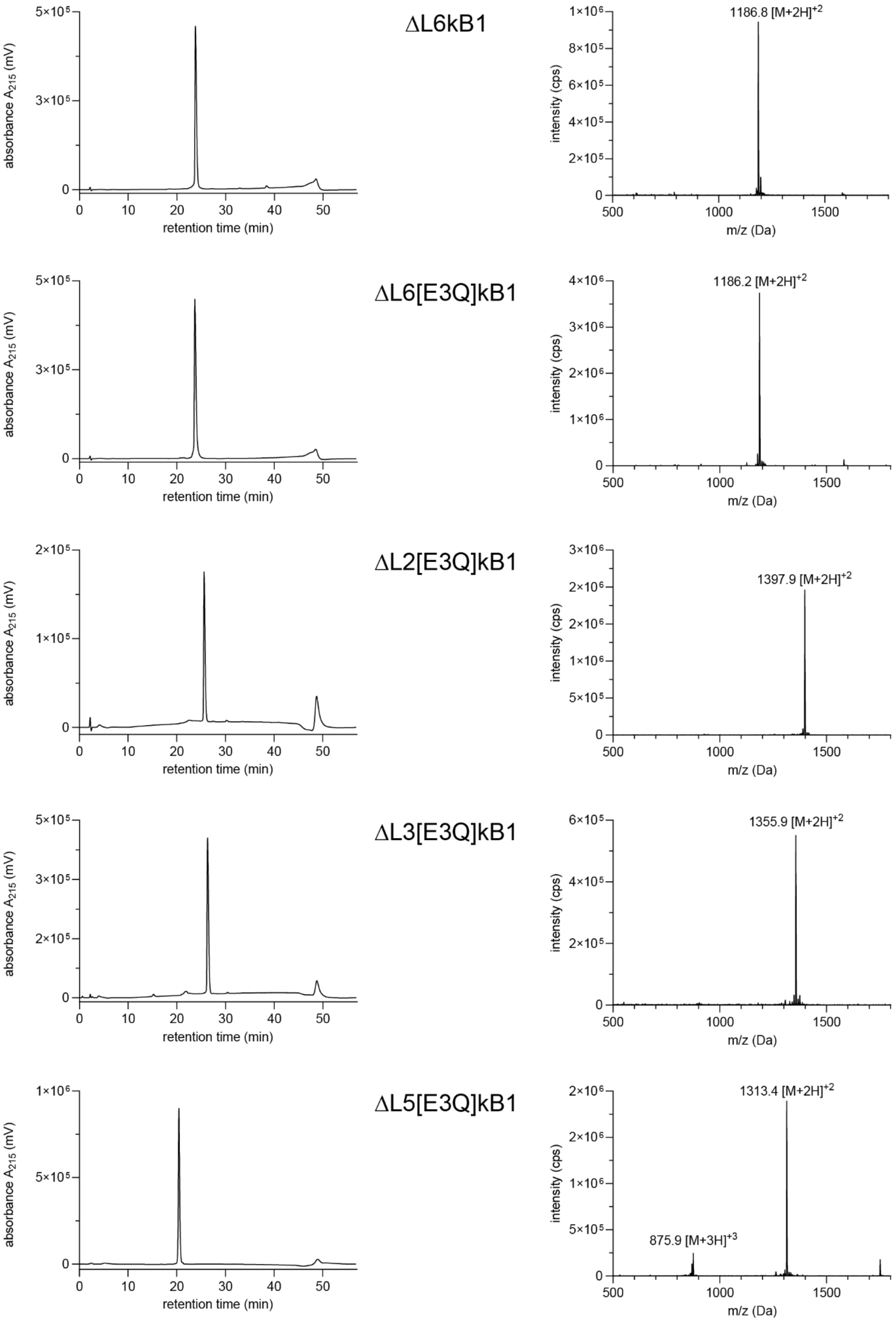

Fig. S2 Analytical HPLC and mass spectra of oxidized scaffold peptides

Section 3: Oxidation and characterization of protected epitope peptides

Peptides containing Acm-protected cysteine residues (Table S2) were dissolved in a 50:50 mixture of solvent A and B. Five equivalents of iodine in methanol were added and the solution stirred at room temperature for 30 minutes. Excess iodine was quenched by adding a solution of ascorbic acid (10 mg/mL in water) until the solution turned colourless. Samples were diluted at least 10 x HPLC solvents prior to loading onto columns.

Table S2. Linear protected epitopes

Peptide	Sequence[a]	Mass (Da)[b]		yield (%)[c]
		calculated	observed	
Boc-L6	Boc-R(Pbf)N(Trt)GLP	1150.3 $[M+H]^{+}$	1150.5 $[M+H]^{+}$	50.9
Boc-dynA	Boc-Y(tBu)GGFLR(Pbf)R(Pbf)IR(Pbf)PK(ivDde)LK(ivDde)	1465.0 $[M+2H]^{+2}$, 977.0 $[M+3H]^{+3}$	1465.4 $[M+2H]^{+2}$, 977.2 $[M+3H]^{+3}$	n.d.[d]
Boc-octreotide	Boc-fC(Acm)Fw(Boc)K(ivDde)T(tBu)C(Acm)T(tBu)	1551.4 $[M+H]^{+}$	1551.7 $[M+H]^{+}$	7.0
Boc-SFTI	Boc-GCT(tBu)K(ivDde)S(tBu)IPPICG	1491.5 $[M+H]^{+}$	1491.8 $[M+H]^{+}$	4.6
Boc-MrIA	Boc-GC(Acm)GY(tBu)K(ivDde)LCG	1160.3 $[M+H]^{+}$	1160.2 $[M+H]^{+}$	8.4
Boc-OT	Boc-GC(Acm)Y(tBu)IQ(Trt)N(Trt)C(Acm)PLG	1705.5 $[M+H]^{+}$	1705.3 $[M+H]^{+}$	36.0
Boc-VP	Boc-GC(Acm)Y(tBu)FQ(Trt)N(Trt)C(Acm)PR(Pbf)G	1017.7 $[M+2H]^{+2}$	1792.3 $[M+H]^{+e}$	35.0
Boc-BM213	Boc-FK(ivDde)PLAAaR(Pbf)	1431.5 $[M+H]^{+}$, 716.3 $[M+2H]^{+2}$	1431.4 $[M+H]^{+}$, 716.4 $[M+2H]^{+2}$	43.3
Boc-BM221	Boc-FK(ivDde)PLA[Abu]aR(Pbf)	1445.5 $[M+H]^{+}$, 723.3 $[M+2H]^{+2}$	1445.5 $[M+H]^{+}$, 723.4 $[M+2H]^{+2}$	50.0

[a]All peptides were synthesized as C-terminal acids

[b]Represents monoisotopic mass of oxidized peptides

[c]Yields were calculated based on initial resin loading

[d]n.d. not determined

[d]Putative fragmentation during ESI-MS, observed m/z corresponding to peptide minus one Trt group (242 Da)

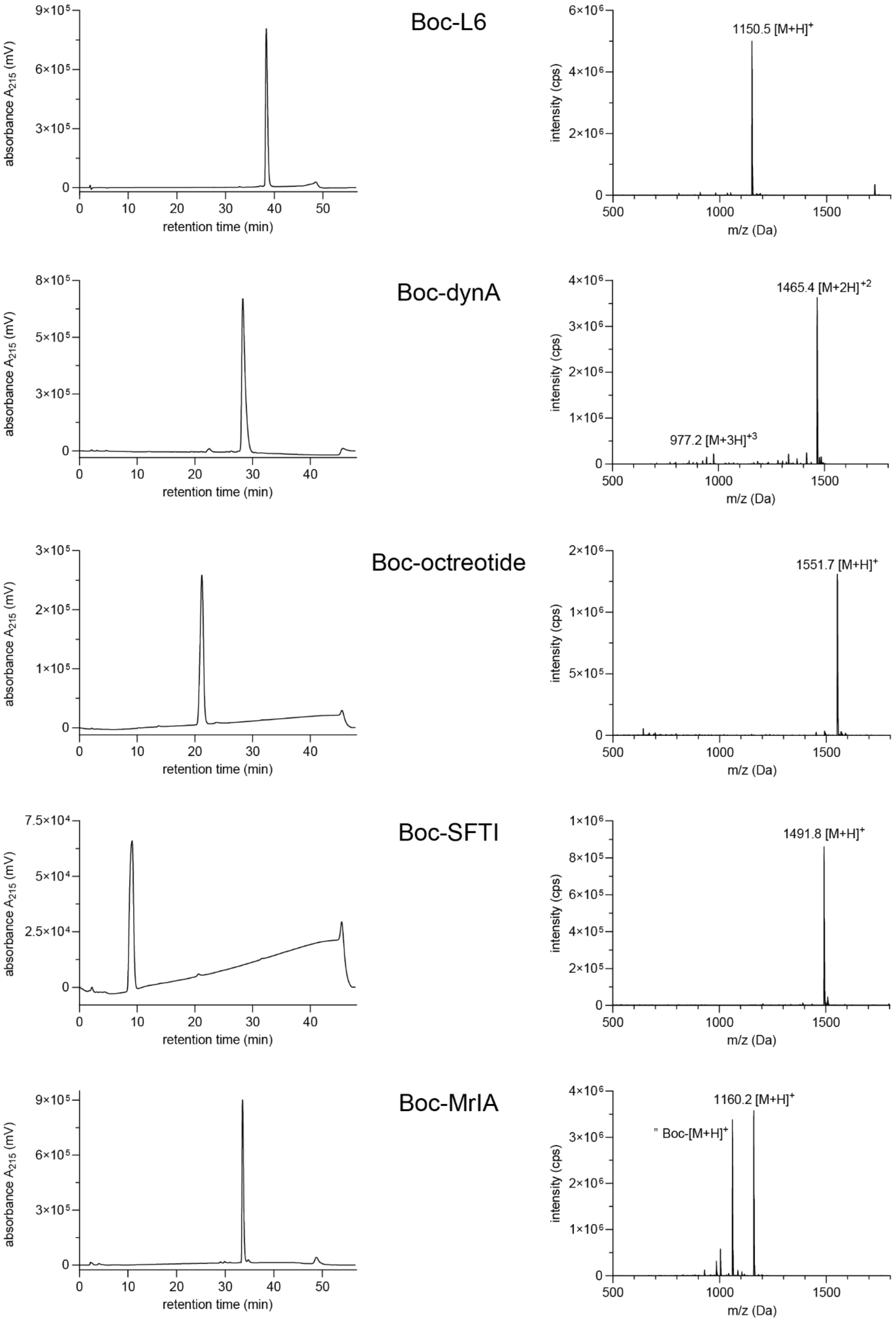

Fig. S3a Analytical HPLC and mass spectra of epitope peptides

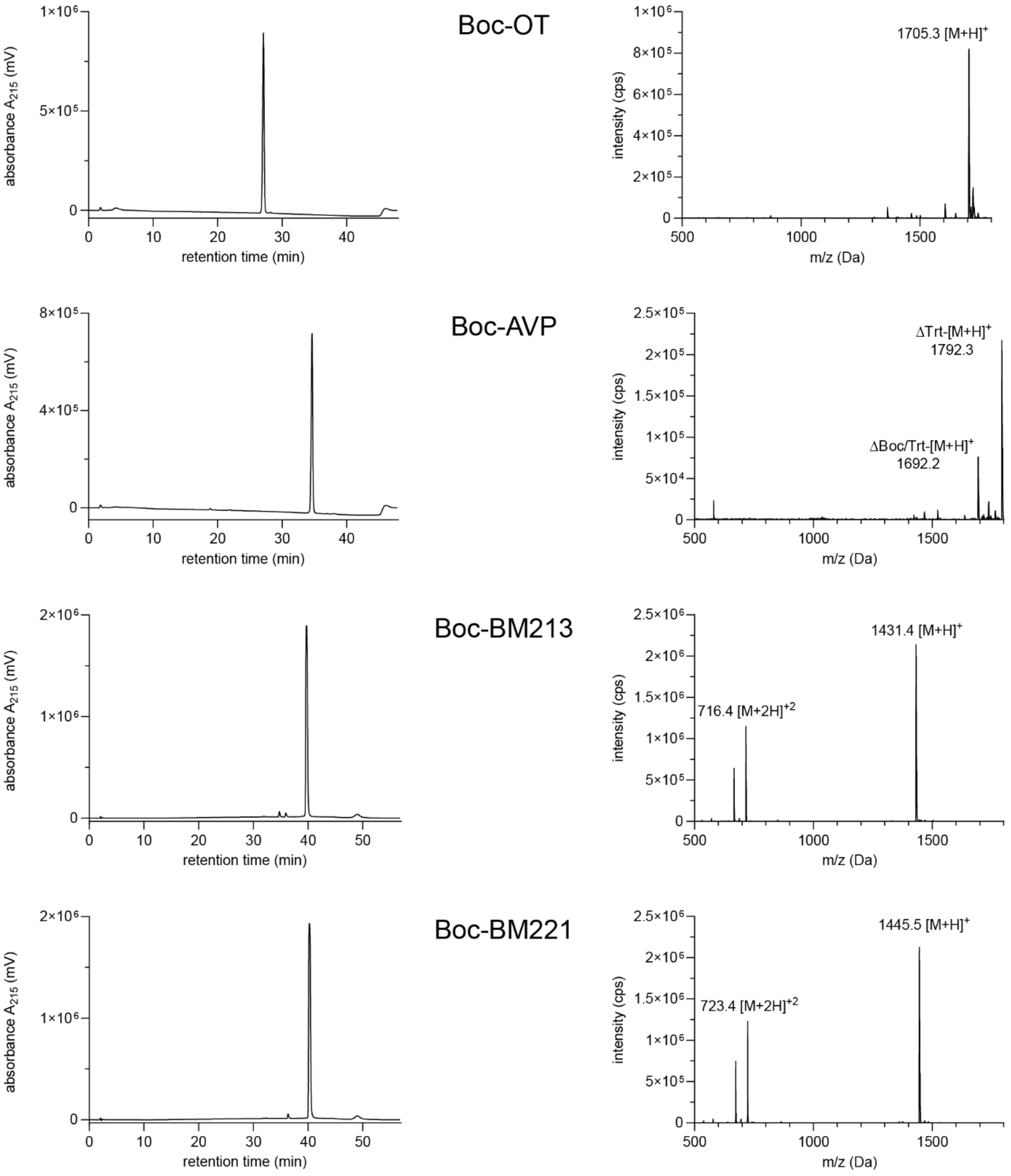

Fig. S3b Analytical HPLC and mass spectra of epitope peptides

Section 4: 'plug and play' grafting and characterization of cyclic peptides

Ligation reactions were performed in DMF by pre-activating the protected epitope for at least 30 seconds using 4 eq of 2-(7-Aza-1-H-benzotriazole-1-yl)-1,1,3,3-tetramethyluronium hexafluorophosphate (HATU). Upon addition of at least 8 eq of DIPEA (added dropwise until solution turned yellow) the mixture was added to purified scaffold peptides to initiate the ligation reaction. For cyclization reactions a 1:1:2 molar ratio of peptide:HATU:DIPEA was used. Both protected as well as fully deprotected peptides were readily dissolved in DMF. Removal of ivDde from Lys residues was achieved by treating the sample with 5% hydrazine in DMF for 10 minutes. Reactions were monitored using UPLC-MS on a Shimadzu Nexera and 2020 LCMS system or by direct injection MS analysis on a SCIEX API2000 mass spectrometer. Samples containing DMF were diluted at least 10 x with HPLC solvents prior to loading onto columns.

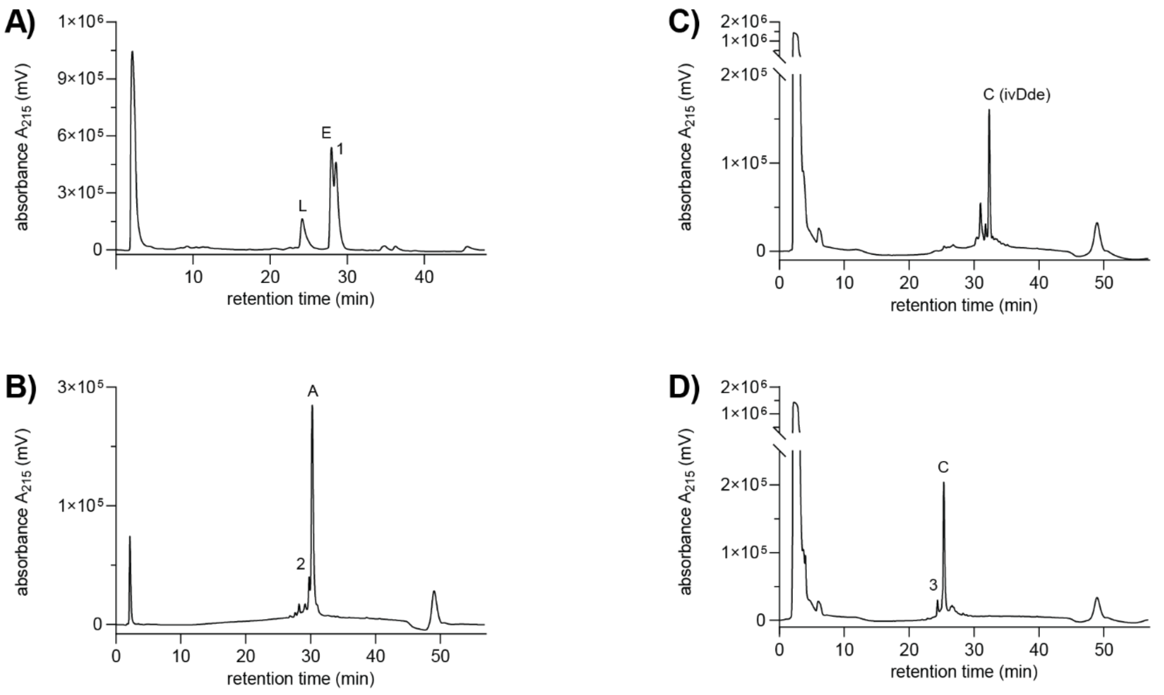

Fig S4. Analytical HPLC monitoring of 'plug and play' grafting strategy of peptide L6-dynA. A) ligation reaction shows excess epitope (E), ligated product (L) and an additional m/z corresponding to the epitope minus one Da (1). B) Deprotection reaction showing acyclic product (A) and additional peak with m/z corresponding to deprotected epitope (2). C) Direct injection MS analysis of cyclization reaction shows one major product with m/z corresponding to cyclic peptide with lysine residues still side-chain protected C(ivDde). D) After hydrazine treatment one major peak with m/z corresponding to fully deprotected cyclic product (C) is observed. One additional peak with m/z corresponding to final product plus 32 Da is identified (3).

Table S3. Plug and play grafted peptides and cyclic controls

Peptide	Sequence[a]	Mass (Da)[b] calculated	Mass (Da)[b] observed	yield (%)[c]
kB1	c-CGETCVGGTCNTPGCTCSWPVCTRNGLPV	1446.1 [M+2H]$^{+2}$	1446.5 [M+2H]$^{+2}$	n.d.[d]
[E3Q]kB1	c-CGQTCVGGTCNTPGCTCSWPVCTRNGLPV	1445.6 [M+2H]$^{+2}$	1446.1 [M+2H]$^{+2}$	23.5/26.1[e]
kB1 p+p	c-CGETCVGGTCNTPGCTCSWPVCTRNGLPV	1446.1 [M+2H]$^{+2}$	1446.6 [M+2H]$^{+2}$	10.6
[E3Q]kB1 p+p	c-CGQTCVGGTCNTPGCTCSWPVCTRNGLPV	1445.6 [M+2H]$^{+2}$	1446.0 [M+2H]$^{+2}$	24.8
ΔL6[E3Q]kB1-dynA	c-YGGFLRRIRPKLKVCGQTCVGGTCNTPGCTCSWPVCT	1313.3 [M+3H]$^{+3}$, 985.2 [M+4H]$^{+4}$, 788.4 [M+5H]$^{+5}$	1313.5 [M+3H]$^{+3}$, 985.5 [M+4H]$^{+4}$, 788.5 [M+5H]$^{+5}$	17.4
ΔL3[E3Q]kB1-dynA	c-YGGFLRRIRPKLKGCTCSWPVCTRNGLPVCGQTCVGGTCN	1426.4 [M+3H]$^{+3}$, 1070.0 [M+4H]$^{+4}$, 856.2 [M+5H]$^{+5}$	1426.5 [M+3H]$^{+3}$, 1070.3 [M+4H]$^{+4}$, 856.3 [M+5H]$^{+5}$	11.2
ΔL5[E3Q]kB1-dynA	c-YGGFLRRIRPKLKVCTRNGLPVCGQTCVGGTCNTPGCTCS	1398.0 [M+3H]$^{+3}$, 1048.8 [M+4H]$^{+4}$, 839.2 [M+5H]$^{+5}$	1398.2 [M+3H]$^{+3}$, 1048.9 [M+4H]$^{+4}$, 839.3 [M+5H]$^{+5}$	7.1
ΔL6[E3Q]kB1-octreotide	c-fCFwKTCTVCGQTCVGGTCNTPGCTCSWPVCT	1684.2 [M+2H]$^{+2}$	1684.3 [M+2H]$^{+2}$	16.1
ΔL6[E3Q]kB1-SFTI	c-GCTKSIPPICGVCGQTCVGGTCNTPGCTCSWPVCT	1704.2 [M+2H]$^{+2}$, 1136.5 [M+3H]$^{+3}$	1704.3 [M+2H]$^{+2}$, 1136.7 [M+3H]$^{+3}$	18.7
ΔL6[E3Q]kB1-MrIA	c-GCGYKLCGVCGQTCVGGTCNTPGCTCSWPVCT	1566.6 [M+2H]$^{+2}$	1566.8 [M+2H]$^{+2}$	18.2
ΔL6[E3Q]kB1-OT	c-GCYIQNCPLGVCGQTCVGGTCNTPGCTCSWPVCT	1700.2 [M+2H]$^{+2}$, 1133.8 [M+3H]$^{+3}$	1700.6 [M+2H]$^{+2}$, 1133. 8 [M+3H]$^{+3}$	10.9
ΔL6[E3Q]kB1-AVP	c-GCYFQNCPRGVCGQTCVGGTCNTPGCTCSWPVCT	1738.7 [M+2H]$^{+2}$, 1159.5 [M+3H]$^{+3}$	1738.4 [M+2H]$^{+2}$, 1159.7 [M+3H]$^{+3}$	1.7
ΔL6[E3Q]kB1-BM213	c-FKPLAAaRVCGQTCVGGTCNTPGCTCSWPVCT	1604.2 [M+2H]$^{+2}$	1604.4 [M+2H]$^{+2}$	22.0
ΔL6[E3Q]kB1-BM221	c-FKPLA(Abu)aRVCGQTCVGGTCNTPGCTCSWPVCT	1611.2 [M+2H]$^{+2}$, 1074.5 [M+3H]$^{+3}$	1611.3 [M+2H]$^{+2}$, 1074.7 [M+3H]$^{+3}$	1.1

[a]c denotes backbone cyclization, all cysteines are oxidized

[b]Represents monoisotopic mass of fully oxidized peptides

[c]Yields were calculated based on initial amount of starting material used for ligation

[d]n.d. not determined – authentic control material obtained from previous work[1]

[e]Yields represent oxidation yields for initial and optimized conditions and are calculated by automated integration of the area under the curve

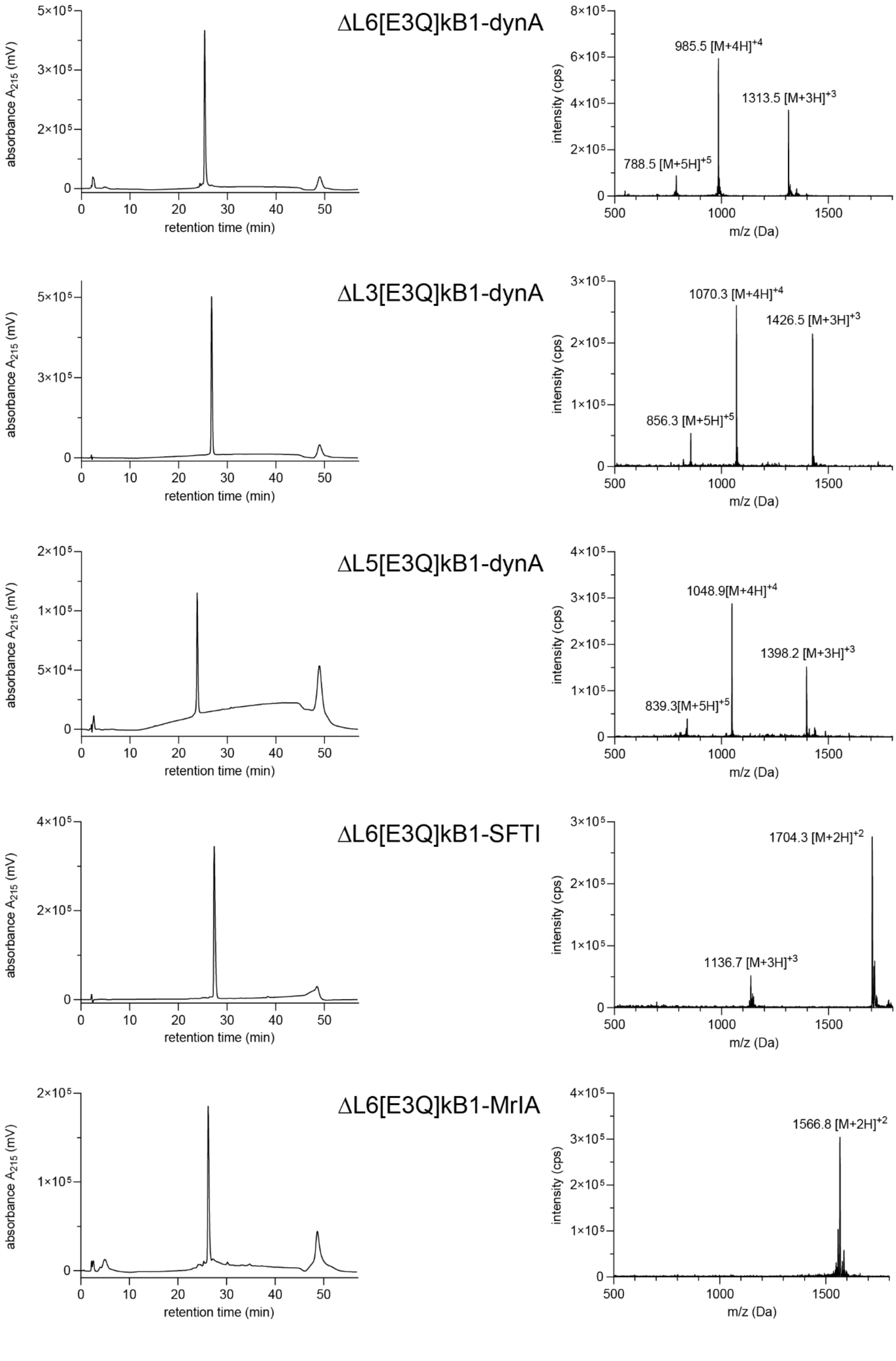

Fig. S5a Analytical HPLC and mass spectra of grafted peptides

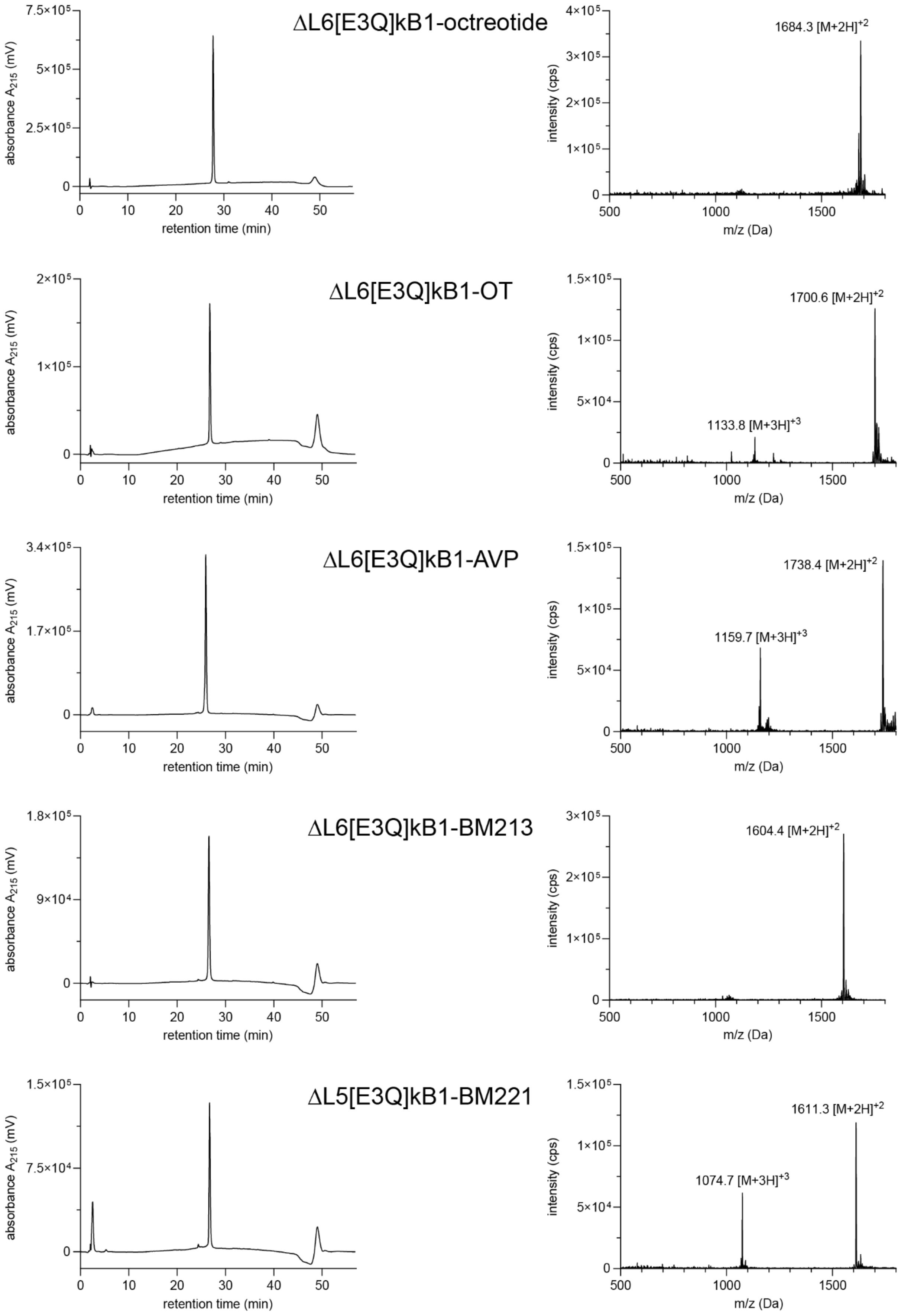

Fig. S5b Analytical HPLC and mass spectra of grafted peptides

Section 5: Carboxypeptidase Y digest and MALDI-TOF MS

Aliquots of peptides (20µL of ~100µM) were reduced for 30 minutes at 60 ^{o}C using DTT (final concentration of 20 mM) and alkylated for 15 minutes at room temperature with iodoacetamide (final concentration of 50 mM). 0.5 units of carboxypeptidase Y were added to fully reduced and alkylated samples and incubated for one hour. Samples were desalted using C18 ZipTips prior to spotting on a MALDI stainless steel plate (mixed 1:1, v/v with a saturated solution of α-cyano-4-hydroxycinnamic acid). Spectra were acquired on a MALDI-TOF/TOF 5800 Analyzer (AB Sciex) operated in reflector positive mode.

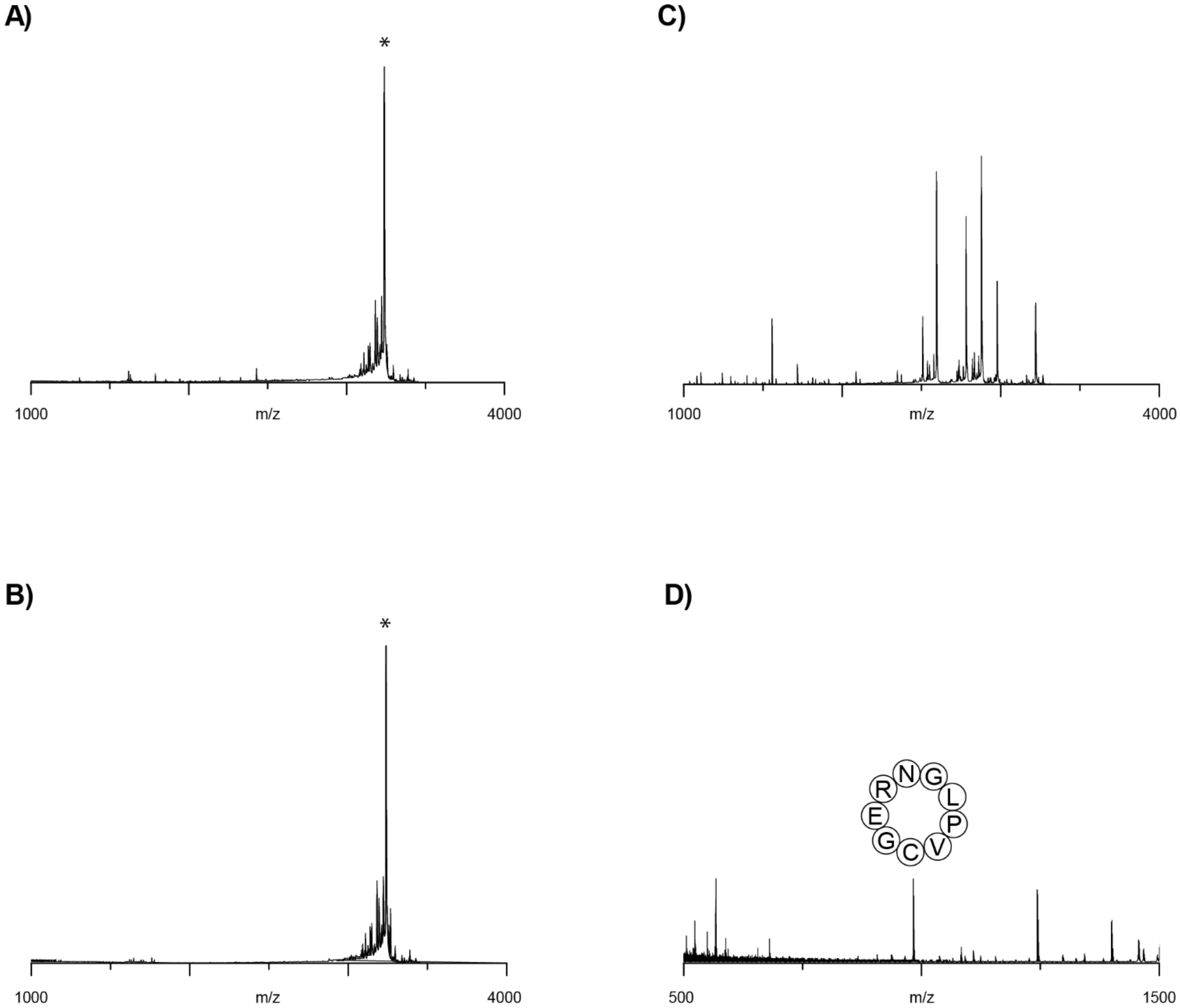

Fig. S6 MALD-TOF MS comparison of native kB1 and the two products obtained from cyclization reaction. After one hour incubation no change in the m/z of fully reduced and alkylated native kB1 (A) and the minor cyclization reaction product (B) is observed (* m/z 3238.0 Da $[M+H]^+$). The major product (C) is readily digested until m/z 982.9 Da $[M+H]^+$ (D), corresponding to the cyclized fragment RNGLPVCGE.

Section 6: KOR pharmacology

HEK293 cells were cultured in Dulbecco's modified Eagle medium (DMEM) containing 10% fetal bovine serum and 50 U/mL penicillin and streptomycin and were grown at 37 °C and 5% CO_2. The HEK293 cell line stably expressing mouse KOR-EGFP was generated by geneticin disulfate (0.8 mg/mL of G418, ROTH, Austria) selection and flow cytometry to select cells for stable cell line propagation. Positive clones were identified by radioligand binding studies as previously described.[2]

Radioligand Competition Binding Assays

Membranes were prepared from HEK293 cells stably expressing the KOR as previously described.[2] Radioligand binding studies were carried out in duplicate using standard binding buffer containing 50 mM Tris−HCl (pH 7.4), 10 mM $MgCl_2$, and 0.1% BSA. For competition binding, 75 µL each of [3H]-diprenorphine (1 nM final), peptide solution (4×), and membrane preparations (7 µg/assay) was incubated in the standard binding buffer, and binding reactions were incubated for 1 h at 37 °C. Termination of reactions was performed by rapid filtration onto a 0.1% polyethylenimine-soaked GF/C glass fiber filter with a Skatron cell harvester.

cAMP Assay

Functional cAMP assay was conducted in triplicate using HEK293 cells stably expressing the mouse KOR according to the manufacturer's protocol with slight modifications. Briefly, 2000 cells per 5 µL per well were seeded into a white 384-well plate and incubated with 5 µL of logarithmically spaced concentrations of peptide solutions prepared (2×) in 1× stimulation buffer and forskolin (10 µM final). The reaction mixture was incubated at 37 °C for 30 min followed by addition of Europium cryptate-labeled cAMP and cAMP d2-labeled antibodies (5 µL of each). After incubation for 1 h at room temperature, cAMP quantification was measured by homogenous time-resolved fluorescence resonance energy transfer on a Flexstation 3 (Molecular Devices, San Jose, USA) using a ratio of 665/620 nm.

Section 7: PRESTO-Tango assay

HTLA cells – a human embryonic kidney cell line (HEK293T) stably expressing β-arrestin2-TEV and tTA-driven luciferase – were maintained in complete Dulbecco's modified Eagle's medium (DMEM) supplemented with 10% of Fetal Bovine Serum, 2.5 µg/mL of puromycin and 50 µg/mL of hygromycin. Approximately 20.000 cells per 100uL per well were seeded in white poly-L-lysine coated 96-well plates and incubated overnight. Transfection of cells was performed using 100 ng of plasmid per well using the calcium phosphate precipitation method and incubation for 24 hours. Medium was then replaced with 100 µL of fresh serum-free DMEM and 50 µL of logarithmically spaced concentrations of peptide (3x stock). Following 24 hours of cell stimulation, medium and drug solutions were removed and 100 µL of homemade Glo reagent was added to each well.[3] After incubation for at least 5 minutes at room temperature luminescence was measured on a Clariostar plate reader.

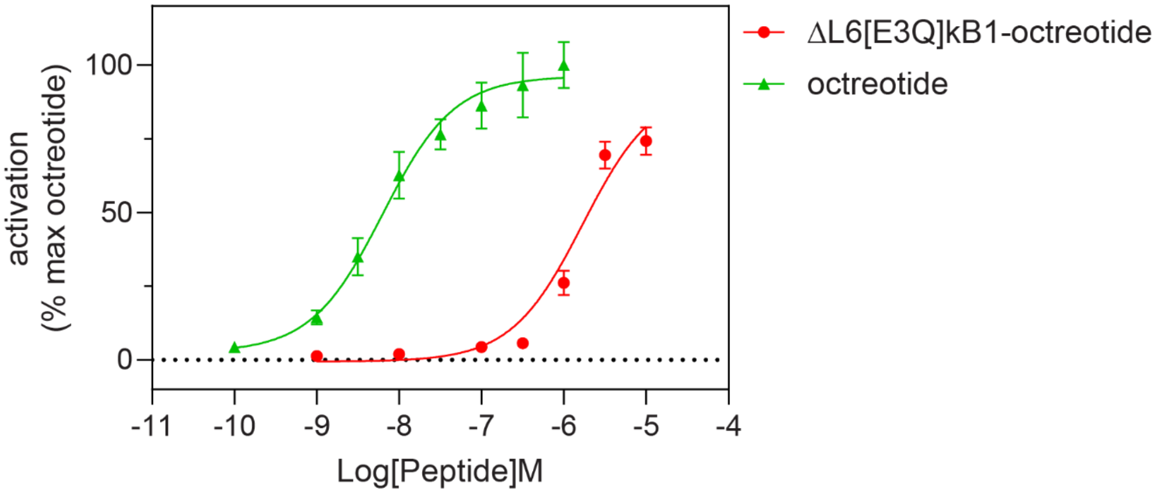

Fig. S7 Activity of grafted ΔL6[E3Q]kB1-octreotide versus the linear epitope octreotide (EC_{50} = 6.9 ± 3.4 nM) using the Presto-Tango assay. Data are mean ± SEM from n=3.

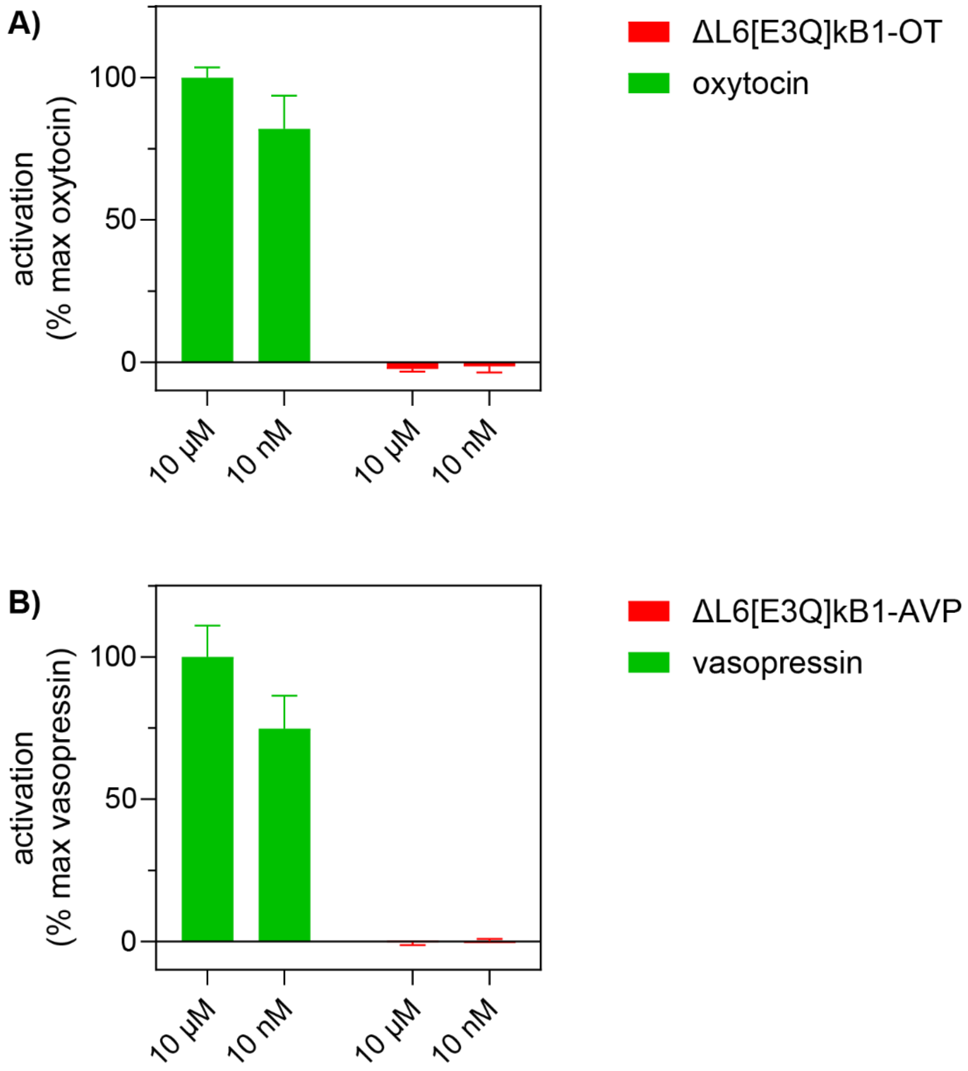

Fig. S8 Activity of grafted ΔL6[E3Q]kB1-OT (A) and ΔL6[E3Q]kB1-AVP (B) versus the linear epitopes oxytocin (OT)/arginine-vasopressine (AVP) using the Presto-Tango assay. Data are mean ± SEM from n=2.

Section 8: pERK assay

Ligand-induced phospho-ERK1/2 signalling was assessed using the AlphaLISA Surefire Ultra p-ERK1/2 (Thr202/Tyr204) kit (PerkinElmer) according to manufactures instructions and as previously described.[4] Chinese Hamster Ovary (CHO) cells, stably expressing human C5aR1, were cultured in Ham's F12 medium supplemented with 10% foetal calf serum (FCS), 100 U/ml penicillin, 100 µg/mL streptomycin, and 400 µg/ml G418 (Invivogen). Upon reaching 80-90% confluency, they were seeded into 96-well plates at a density of 50,000 cells/well and serum-starved overnight. Ligand dilutions were prepared in serum-free medium, and cells were incubated with peptide ligands for 10 min at room temperature and then immediately lysed using AlphaLISA lysis buffer on a microplate shaker (450 rpm, 10 min). For the detection of phospho-ERK1/2 content, cell lysate (5 µL/well) was transferred to a 384-well ProxiPlate (PerkinElmer) and added to the donor and acceptor reaction mix (2.5 µL/ well, respectively). After two hours of incubation at room temperature in the dark, the plate was read Tecan Spark 20M following standard AlphaLISA settings.

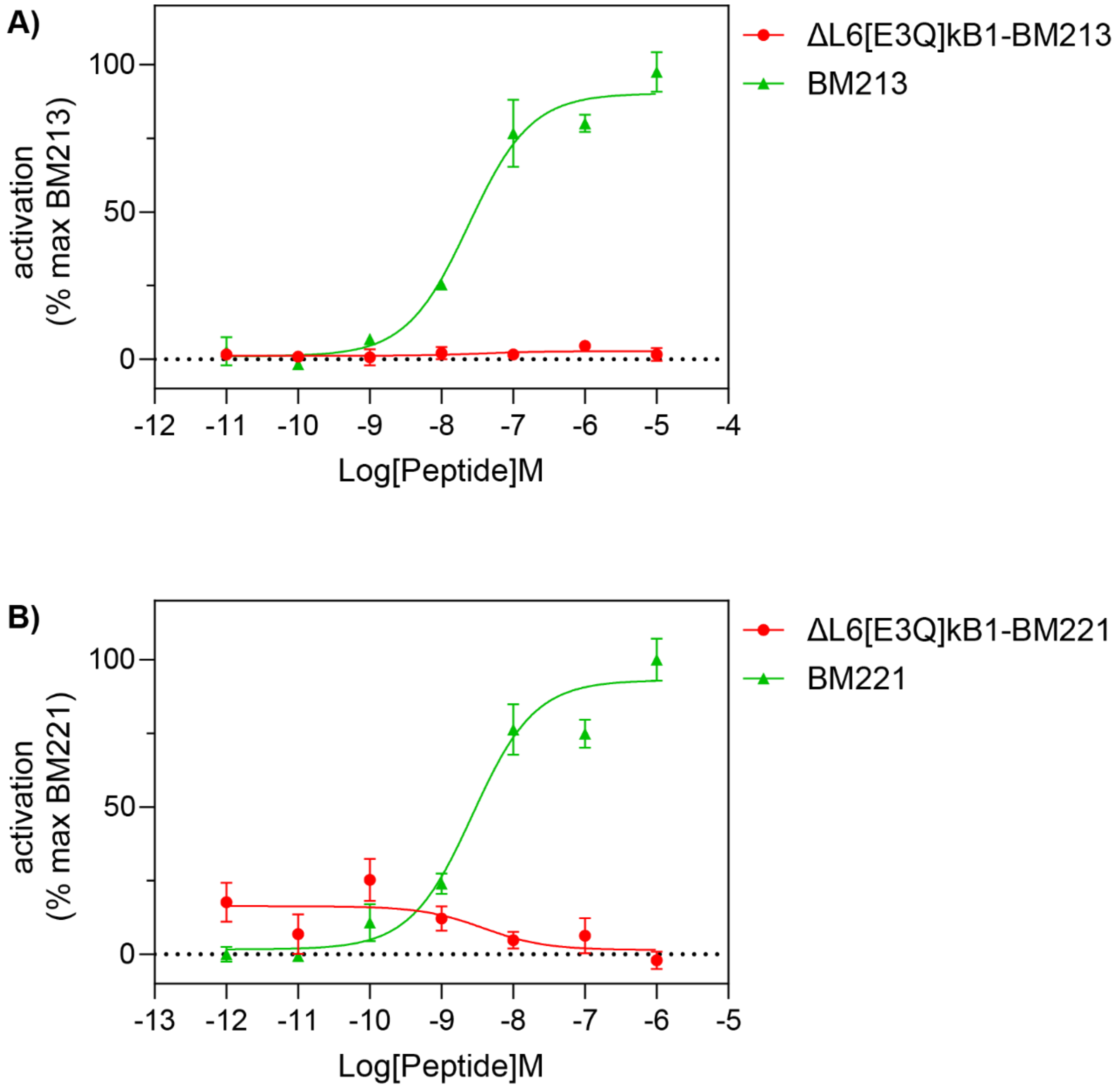

Fig. S9 Activity of grafted ΔL6[E3Q]kB1-BM213 (A) and ΔL6[E3Q]kB1-BM221 (B) versus the linear epitopes BM213 (EC_{50} = 38.6 ± 17.2 nM) and BM221 (EC_{50} = 2.8 ± 0.9 nM) using the pERK assay. Data are mean ± SEM from n=2.

Section 9: Trypsin inhibition assay

The inhibition was determined as previously described.[5] kB1-SFTI-L6 were dissolved in water at stock concentration of 1 mM. The assay buffer was 50 mM Tris-HCl, pH 7.8 containing 20 mM calcium chloride. 20 µL of 25 µg/mL trypsin from bovine pancreas (Sigma-Aldrich) and 5 µL of increasing concentrations of peptide (giving final concentrations of 0−16.6 µM) was pre-incubated at 37 °C for 15 min. The reaction was initiated by adding 125 µL of 1 mM N-α-benzoyl-L-arginine-p-nitroanilide substrate (Sigma-Aldrich) and incubated for 30 min at 37 °C. Synthetic SFTI was used as positive control. Reactions were performed in triplicate. Wells with no peptides were designated 100% trypsin activity. To stop the reactions, 25 µL of 30% acetic acid was added and absorbance was measured at 410 nm.

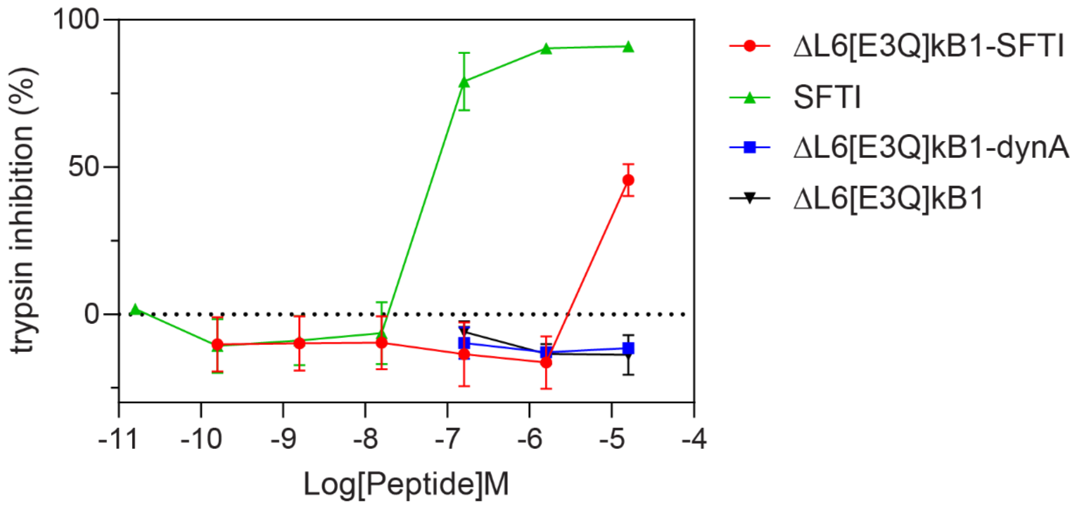

Fig. S10 Trypsin inhibition of grafted ΔL6[E3Q]kB1-SFTI vs native SFTI. Data are mean ± SEM from n=3. No trypsin inhibition was observed with the linear scaffold peptide ΔL6[E3Q]kB1 and another graft, i.e. ΔL6[E3Q]kB1-dynA.

Section 10: NMR characterization

Samples were prepared by dissolving ~0.5–1 mg of dry peptide in 0.5 mL of 90% H2O/10% D2O (vol/vol). NMR experiments, including 1D, total correlation spectroscopy (TOCSY) and nuclear Overhauser effect spectroscopy (NOESY), were recorded on a Bruker Avance-600 MHz spectrometer at 298 K. Solvent suppression was achieved using pulse sequences with excitation sculpting (zgesgp, mlevesgpph and noesyesgpph). TOCSY and NOESY experiments were acquired with a mixing time of 80 and 200 ms respectively, 4,096 complex data points in the F2 dimension and 512 increments in the F1 dimension, and they were processed using TopSpin (Bruker Biospin) software. Chemical shifts were referenced to internal 2,2-dimethyl-2-silapentane-5-sulfonate. Spectra were analyzed in CCPNMR software.[6]

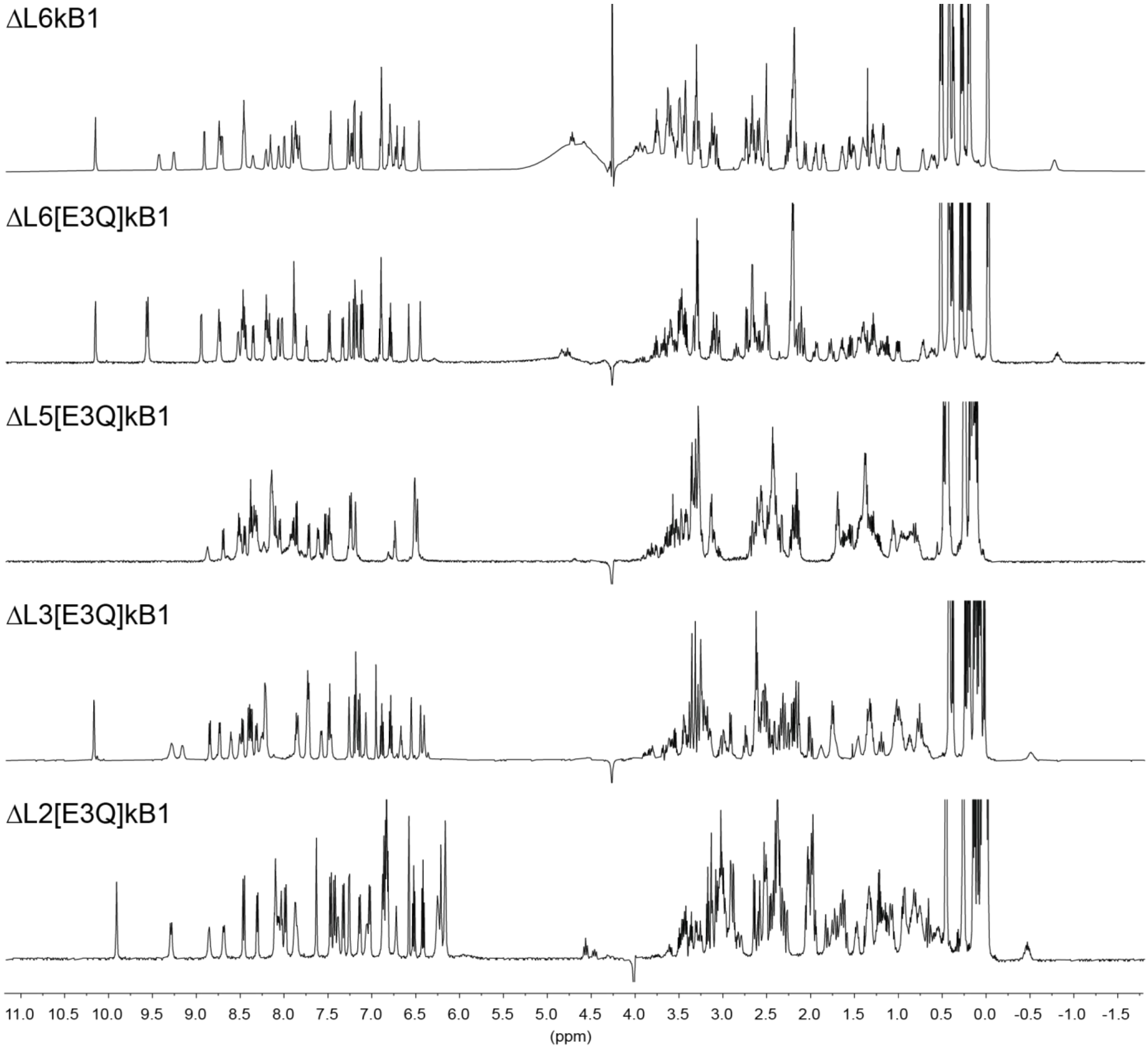

Fig. S11 ^{1}H NMR spectra of linear oxidized scaffold peptides (acquired at 600MHz, solvent H_2O/D_2O (9:1)

References

1 O. Cheneval, C. I. Schroeder, T. Durek, P. Walsh, Y. H. Huang, S. Liras, D. A. Price and D. J. Craik, *J. Org. Chem.*, 2014, **79**, 5538-5544.

2 E. Muratspahić, N. Tomašević, J. Koehbach, L. Duerrauer, S. Hadžić, J. Castro, G. Schober, S. Sideromenos, R. J. Clark, S. M. Brierley, D. J. Craik and C. W. Gruber, *J. Med. Chem.*, 2021, **64**, 9042-9055.

3 M. Zeghal, G. Laroche and P. M. Giguère, *J. Vis. Exp.*, 2020, DOI: 10.3791/60823.

4 D. M. Gorman, X. X. Li, J. D. Lee, J. N. Fung, C. S. Cui, H. S. Lee, B. E. Rolfe, T. M. Woodruff and R. J. Clark, *J. Med. Chem.*, 2021, **64**, 16598-16608.

5 J. Zhang, C. D. Payne, B. Pouvreau, H. Schaefer, M. F. Fisher, N. L. Taylor, O. Berkowitz, J. Whelan, K. J. Rosengren and J. S. Mylne, *ACS Chem. Biol.*, 2019, **14**, 979-993.

6 S. P. Skinner, R. H. Fogh, W. Boucher, T. J. Ragan, L. G. Mureddu and G. W. Vuister, *J. Biomol. NMR*, 2016, **66**, 111-124.
